# Relationship between fine particulate matter, weather condition and daily non-accidental mortality in Shanghai, China: A Bayesian approach

**DOI:** 10.1371/journal.pone.0187933

**Published:** 2017-11-09

**Authors:** Xin Fang, Bo Fang, Chunfang Wang, Tian Xia, Matteo Bottai, Fang Fang, Yang Cao

**Affiliations:** 1 Unit of Biostatistics, Institute of Environmental Medicine, Karolinska Institutet, Stockholm, Sweden; 2 Division of Vital Statistics, Shanghai Municipal Center for Disease Control and Prevention, Shanghai, China; 3 Department of Environmental Health, School of Public Health, Fudan University, Shanghai, China; 4 Institute of Health Information, Shanghai Municipal Center for Disease Control and Prevention, Shanghai, China; 5 Department of Medical Epidemiology and Biostatistics, Karolinska Institutet, Stockholm, Sweden; 6 Clinical Epidemiology and Biostatistics, School of Medical Sciences, Örebro University, Örebro, Sweden; The Ohio State University, UNITED STATES

## Abstract

There are concerns that the reported association of ambient fine particulate matter (PM_2.5_) with mortality might be a mixture of PM_2.5_ and weather conditions. We evaluated the effects of extreme weather conditions and weather types on mortality as well as their interactions with PM_2.5_ concentrations in a time series study. Daily non-accidental deaths, individual demographic information, daily average PM_2.5_ concentrations and meteorological data between 2012 and 2014 were obtained from Shanghai, China. Days with extreme weather conditions were identified. Six synoptic weather types (SWTs) were generated. The generalized additive model was set up to link the mortality with PM_2.5_ and weather conditions. Parameter estimation was based on Bayesian methods using both the Jeffreys’ prior and an informative normal prior in a sensitivity analysis. We estimate the percent increase in non-accidental mortality per 10 μg/m^3^ increase in PM_2.5_ concentration and constructed corresponding 95% credible interval (CrI). In total, 336,379 non-accidental deaths occurred during the study period. Average daily deaths were 307. The results indicated that per 10 μg/m^3^ increase in daily average PM_2.5_ concentration alone corresponded to 0.26–0.35% increase in daily non-accidental mortality in Shanghai. Statistically significant positive associations between PM_2.5_ and mortality were found for favorable SWTs when considering the interaction between PM_2.5_ and SWTs. The greatest effect was found in hot dry SWT (percent increase = 1.28, 95% CrI: 0.72, 1.83), followed by warm humid SWT (percent increase = 0.64, 95% CrI: 0.15, 1.13). The effect of PM_2.5_ on non-accidental mortality differed under specific extreme weather conditions and SWTs. Environmental policies and actions should take into account the interrelationship between the two hazardous exposures.

## Introduction

Both extreme weather conditions and particulate matter air pollution are well-established risk factors of adverse health outcomes. There is a wealth of evidence showing that all-cause mortality increases during the cold season [1–16]. Exposure to high ambient temperature has also been associated with increased mortality in many countries [17–25]. In view of the worldwide climate change, the health effects of both hot and cold weathers are becoming a global public challenge for the 21st century [26]. Particulate matter air pollution, especially particulate matter 2.5 micrometers or less in diameter (PM_2.5_), or fine particulate matter, is another main contributor to premature mortality [27, 28]. It has long been recognized that particle concentrations are correlated with mortality both temporally (short-term fluctuations) and spatially [29, 30]. PM_2.5_ has been one of the major causes of premature mortality in Asia, Europe and America [31–36]. According to the Air quality in Europe—2015 report, about 432,000 premature deaths were attributable to PM_2.5_ exposure in 2012 in 40 European countries [37]. A recent study indicated that an aggressive global program of PM_2.5_ mitigation in line with the World Health Organization (WHO) interim guidelines could avoid almost one fourth of the deaths attributable to ambient PM_2.5_ [38]. Understanding the relationship between acute exposure to PM_2.5_ and mortality is therefore critical.

Although it is well documented that daily non-accidental mortality fluctuations are positively and significantly associated with PM_2.5_ and meteorological conditions, whether meteorological conditions confound or modify the association of the air pollutant with mortality is rarely investigated. There are concerns that the reported association of PM_2.5_ with mortality might be a mixture of PM_2.5_ and weather conditions [28, 39–43].

Furthermore, most of the existing epidemiological studies used population-level aggregated data and lacked individual-level information on potentially important behavioral and socioeconomic factors, leading to potential concerns of confounding [44, 45]. In time series studies, generalized additive model (GAM) and generalized additive mixed model (GAMM) have been widely applied because they may control for the nonlinear confounding effects of seasonal trend and meteorological variables [46–53], in addition to the fact that they are more flexible than fully parametric alternatives. To control for the weather conditions in estimating the independent effect of air pollution on mortality, the usual analytic strategies are either including meteorological variables in regression models or using time-stratified approach to investigate the season-specific effects of pollutants on mortality [39, 54]. Few studies have however explored the interaction between particulate matters and meteorological variables, which leaves the form and possible mechanisms of the interaction largely unknown [55–57]. The models might also yield biased estimates of the effects of air pollutants on mortality when inappropriate meteorological variables were used or when the models failed to properly reflect the underlying weather-mortality association [39]. Although conventional GAM or GAMM gives a rich family of models that have been widely applied, in terms of analytical tractability, inference is dependent on asymptotic sampling distributions of estimators. So far few guidelines are available as to when such theory will produce accurate inference and the degree to which inference is dependent on modeling assumptions is unknown [58]. A Bayesian approach is attractive in this case. Under a probability model, it provides inferences that are conditional on the data and are exact, without reliance on asymptotic approximation. A Bayesian approach also provides interpretable answers, such as “the true parameter has a probability of 0.95 of falling in a 95% credible interval (CrI)” [59, 60].

In our time series study, we applied GAM for fitting and inference within a Bayesian framework to explore the associations of mortality with PM_2.5_ and weather. We examined the effects of extreme weather conditions and weather types on mortality as well as their interactions with PM_2.5_ concentrations. We also estimate the percent increase for non-accidental mortality attributable to PM_2.5_ exposure and weather conditions, adjusted for individual-level and contextual covariates, including sex, age, smoking and occupation.

The study was approved by the Ethical Review Committee of the Shanghai Municipal Center for Disease Control and Prevention (SCDC), Shanghai China (approval number: 2016–08).

## Materials and methods

### Study setting

The study area is Shanghai, one of the most populous cities in the world, located in the Yangtze River Delta in East China and the middle portion of the Chinese coast. It is served as the most influential economic, financial, international trade, and cultural center in East China. It is also one of the global financial centers and transport hubs, with the world's busiest container port by both volume of the shipment and cargo tonnage. There are 16 administrative districts in Shanghai, all with own urban cores, and the average population for the study period from 2012 to 2014 was 24 million [61].

### Data collection

Daily average PM_2.5_ concentrations between January 1st, 2013 and December 31st, 2014 were obtained from the Shanghai Meteorological Bureau. Only the measurements from one air quality monitor were available during the study period and used for whole Shanghai area. Because PM_2.5_ was not routinely monitored in Shanghai until late 2012, we obtained 2012 data from the published hourly PM_2.5_ concentrations by the air quality monitoring station of the U.S. Consulate General in Shanghai, China, which is located in the Xuhui district of Shanghai. Recent studies have indicated that PM_2.5_ data from the U.S. embassy and consulates’ air quality monitoring stations were highly consistent [62, 63] with the data from Chinese national monitoring stations in the urban districts. The daily average PM_2.5_ concentrations in 2012 were calculated from the hourly concentrations. The daily mortality data during the corresponding time period for all the 16 administrative districts in Shanghai were obtained from the Causes of Death Registry of Shanghai Municipal Center for Disease Control and Prevention (SCDC). The causes of death were coded according to the International Disease Classification Codes, version 10 (ICD-10). Deaths for all non-accidental causes (ICD-10 codes: A00-R99) were examined. Individual information of age, sex, occupation, education, residential area and smoking rates of every 5-year age groups were also obtained from SCDC. Citywide daily meteorological data including temperature, relative humidity, barometric pressure, wind speed, precipitation and sunshine time were retrieved from the Shanghai Meteorological Bureau as well and no district-specific data available in current study.

### Statistical models

Days with extreme weather conditions were identified according to the Guidelines on Analysis of Extremes in a Changing Climate in Support of Information Decision for Adaptation of the World Meteorological Organization (Climate Data and Monitoring, WCDMP-No. 72) [64]. The indices (i.e. day-count of extremes) concept involves calculation of the number of days in a year exceeding specific thresholds. Examples of such “day-count” indices are the number of days with minimum temperature below the 10th percentile in a given period. We adopted the similar rule to define the eight extreme weather conditions, i.e. hot, cold, hyperbaria, hypobaria, humid, dry, windy and windless, as the daily minimum/maximum temperature, minimum/maximum barometric pressure, average humidity or wind speed lower or higher than the corresponding yearly 10th percentile or 90th percentile in the 3-year study period, respectively.

Because extreme weather conditions are not mutually exclusive, to better investigate the interaction between PM_2.5_ and weather conditions, we categorized the observed days into synoptic weather types (SWTs) as proposed by Kalkstein et al. [65]. This approach categorizes weather patterns using clustering technique and offers categories that represent groupings of meteorological variables as they actually occur at a locale [39]. The statistical methods used have been detailed in previous studies [66–68]. In brief, a set of routinely monitored meteorological parameters (three barometric pressure parameters, three temperature parameters, two humidity parameters, one precipitation parameter, five wind speed parameters and one sunshine parameter) were used for clustering. To reduce the inter-correlation between meteorological parameters, the principal component analysis (PCA) was performed before clustering and generated six uncorrelated principal components (PCs), which explained 93% variance of the original 15 meteorological parameters. The K-means clustering method was used afterwards to categorize the 1096 observed days into the six SWTs based on the six PCs. The number of clusters was decided by the elbow method.

The GAM was set up to link the mortality with PM_2.5_ and weather conditions and can be expressed as:

$$\log\left(E(Y_{t})\right)=\beta_{0}+\beta_{1}∙{PM}_{2.5,t}+\beta_{2}∙W_{t}+\beta_{3}∙{PM}_{2.5,t}\times W_{t}+\beta_{4}∙Sex+\beta_{5}∙Age+\beta_{6}∙Job+\beta_{7}∙{DOW}_{t}+\beta_{8}∙Smoking+S(t)$$ (1)

where *E*(*Y*_*t*_) refers to the expected count of deaths on day *t*; *PM*_2.5,*t*_ refers to the PM_2.5_ concentration on day *t*; ***W***_***t***_ = (*W*_1_,⋯,*W*_*j*_)′ denotes a *j*×1 vector (*j* = 5 or 7) of *j* dummy variables of the six SWTs or the eight extreme weather conditions on day *t*; *PM*_2.5,*t*_ × ***W***_*t*_ denotes the interaction term between PM_2.5_ and ***W***_*t*_; *Sex* is a dummy variable of sex; ***Age*** denotes a 3×1 vector of three dummy variables of four age categories (0–14, 15–39, 40–64 and 65+ years); ***Job*** denotes a 10×1 vector of ten dummy variables of 11 occupation categories; ***DOW***_*t*_ denotes a 6×1 vector of six dummy variables of day of week (DOW) for day *t*; Smoking denotes smoking rate; *S*(·) is the smoothing function realized by cubic B-splines. Based on generalized cross-validation and our simulation study, which indicated that 14 knots were enough to present the temporal trend and capture the underlying true parameter of PM2.5, we used in total 15 knots (5 knots per year) for the splines, which were corresponding to the largest likelihood. Effects from unobserved confounders and seasonal trend of meteorological variables were presented by the smoothing function. In the model, the subgroups with the most deaths were set as reference groups (except for sex and DOW). To make a comparison, the models without interaction term were also examined in the study.

Daily mortality *Y* follows a Poisson distribution, i.e. *Y* ~ Poisson (*μ*(***X***)), where the log-mean parameter is the linear function shown in (1). For a given input vector ***X***_*t*_ we have $\mu (X_{t})=e^{\beta_{0}+X_{t}^{T}\beta +S(t)}$. Depending on this parameterization, the probability of an outcome *Y*_*t*_ given ***X***_*t*_ is:

$$p(Y_{t}|X_{t},\beta ,S,t)=e^{-e^{\beta_{0}+X_{t}^{T}\beta +S(t)}}\frac{e^{[\beta_{0}+X_{t}^{T}\beta +S(t)]Y_{t}}}{Y_{t}!}$$ (2)

and thus the likelihood for a training data ***X*** and *Y* is:

$$L(Y|X,\beta ,S)=\prod_{t=1}^{N}\left\{\frac{e^{-e^{\beta_{0}+X_{t}^{T}\beta +S(t)}}e^{{[\beta }_{0}+X_{t}^{T}\beta +S(t){]Y}_{t}}}{Y_{t}!}\right\}$$ (3)

According to the Bayes' theorem, the posterior distribution of the parameters proportionates to the production of the prior distribution and the likelihood. Although the posterior distribution can be derived by a distribution approximation method [69], we used the data-driven numerical Markov chain Monte Carlo (MCMC) method to approximate the posterior distribution. To benefit from Bayesian framework with as limited influence from the prior distribution as possible, the Jeffreys’ prior distribution was used for our parameters. Based on the observed Fisher information matrix, Jeffreys’ prior is useful because it does not change much over the region in which the likelihood is significant and does not have large values outside that range–the local uniformity property. Thus, it provides an automated way of finding a non-informative prior for any parametric model. Detailed introduction and discussion about Jeffreys’ prior for GAM have been described elsewhere [70–72].

The key step of Bayesian inference is to reallocate credibility across parameter values, i.e. approximating the posterior distribution of the parameter from the predefined prior distribution to values that are consistent with the data. We used the adaptive rejection sampling algorithm, a type of MCMC method, to sample parameters sequentially from their univariate full conditional distributions [73, 74]. The method may generate samples from an arbitrary posterior density *p*(*β*_*i*_|*y*) of a complex model and to use these samples to approximate expectations of parameters of interest [75]. When log-concavity condition is not met, an additional Metropolis-Hastings step will be applied, and the modified algorithm becomes the adaptive rejection. The Metropolis-Hastings sampling (ARMS) algorithm, however, could have a high computational cost. Implementation of the ARMS algorithm in our study is based on the code provided by Gilks.[76] We set the number of burn-in iterations to 1000 before the Markov chains were saved and the number of iterations after burn-in to 5000 to reduce computation time. Our preliminary experiments showed that the differences of posterior parameter *β*_*i*_s between the 5000 iterations and 100000 iterations were undetectable, but the computation time of 5000 iterations was reduced from more than 10 hours to less than 30 minutes on the computer with 64-bit Windows 7 Enterprise operation system (Service Pack 1), Intel ® Core ™ i7-3687U @ 2.10 GHz CPU and 16.0 GB installed random access memory. The thinness of the Markov chains was set to 10.

Convergence of Markov chains was assessed using Gelman-Rubin method [77, 78]. If the Gelman-Rubin statistic is smaller than 1.01 or so, we define that the chains have converged adequately. Representative of the chains was evaluated visually using the trace plots. If the chains that have been sampled with independent pseudo-random numbers meandered smoothly and overlapped with each other, it means that they are representative [59]. The dependency and efficiency of the MCMC was evaluated using autocorrelation and effective sample sizes (ESS), respectively. Low correlations can indicate good mixing and an ESS of approximately 1000 is adequate for estimating the posterior density [79].

We reported the posterior mean and posterior CrI *A*_*i*_ of *β*_*i*_ in the present paper. The definition of posterior mean is given by:

$$E(\beta_{i}|X,Y,S)=\int \beta_{i}p(\beta_{i}|X,Y,S)d\beta_{i}$$ (4)

where *p*(*β*_*i*_|***X***,*Y*,***S***) is posterior probability of *β*_*i*_ given the observed data ***X*** and *Y*. The definition of posterior CrI *A*_*i*_ is given by:

$$P(\beta_{i}\in A_{i}|X,Y,S)=\int_{A_{i}}p(\beta_{i}|X,Y,S)d\beta_{i}$$ (5)

We constructed a 95% CrI with equal tails corresponding to the 2.5th and 97.5th percentiles of the posterior distribution. The interval is preferred because it is invariant under transformations [60].

Bayesian inference for GAM was performed in SAS 9.4 M4 (SAS Institute Inc, Cary, North Carolina, USA). Smoothing splines were generated by Stata 14.2 (StataCorp LLC, College Station, Texas, USA). Statistical graphing were achieved using SAS and R 3.33 base package (R Foundation for Statistical Computing, Vienna, Austria) and ggplot2 package[80].

## Results

### Demographic characteristics of the subjects

In total, 336,379 non-accidental deaths occurred during the study period between January 1st, 2012 and December 31st, 2014 in Shanghai. Average daily deaths were 307. The demographic characteristics of the subjects are shown in Table 1. The average age of the subjects was 77 years, including 53% males. More than one third (36.86%) of the subjects were from manufactory occupations. The age standardized smoking rate in males was 29.71%, and in females 0.92%.

**Table 1:** Demographic characteristics of the subjects who died during the study period.

Sex, n (%)	
Male	178,786 (53.15%)
Female	153,593 (46.85%)
Age (year), mean±SD	77.0±12.6
Age distribution, n (%)	
0–14 years	1,252 (0.37%)
15–39 years	3,080 (0.92%)
40–64 years	54,404 (16.17%)
65+ years	277,643 (82.54%)
Education, n (%)	
Illiterate	84,943(25.25%)
Preliminary school	100,194 (29.79%)
High school	118,235 (35.15%)
Undergraduate and above	27,063 (8.05%)
NA	5,944 (1.77%)
Occupation, n (%)	
Governmental	2,760 (0.82%)
Professional	28,992 (8.62%)
Administrative	34,431 (11.13%)
Business	32,823 (9.76%)
Agriculture and stockbreeding	77,832 (23.14%)
Manufactory	123,998 (36.86%)
Military	201 (0.06%)
Others	3,185 (0.95%)
Preschooler	1,060 (0.32%)
Students	337 (0.10%)
Retired or jobless	27,760 (8.25%)
Smoking rate ^a^, %	
Male	29.71%
Female	0.92%

^a^ Indirectly standardized rate.

### PM_2.5_ level and meteorological conditions

**Fig 1:**
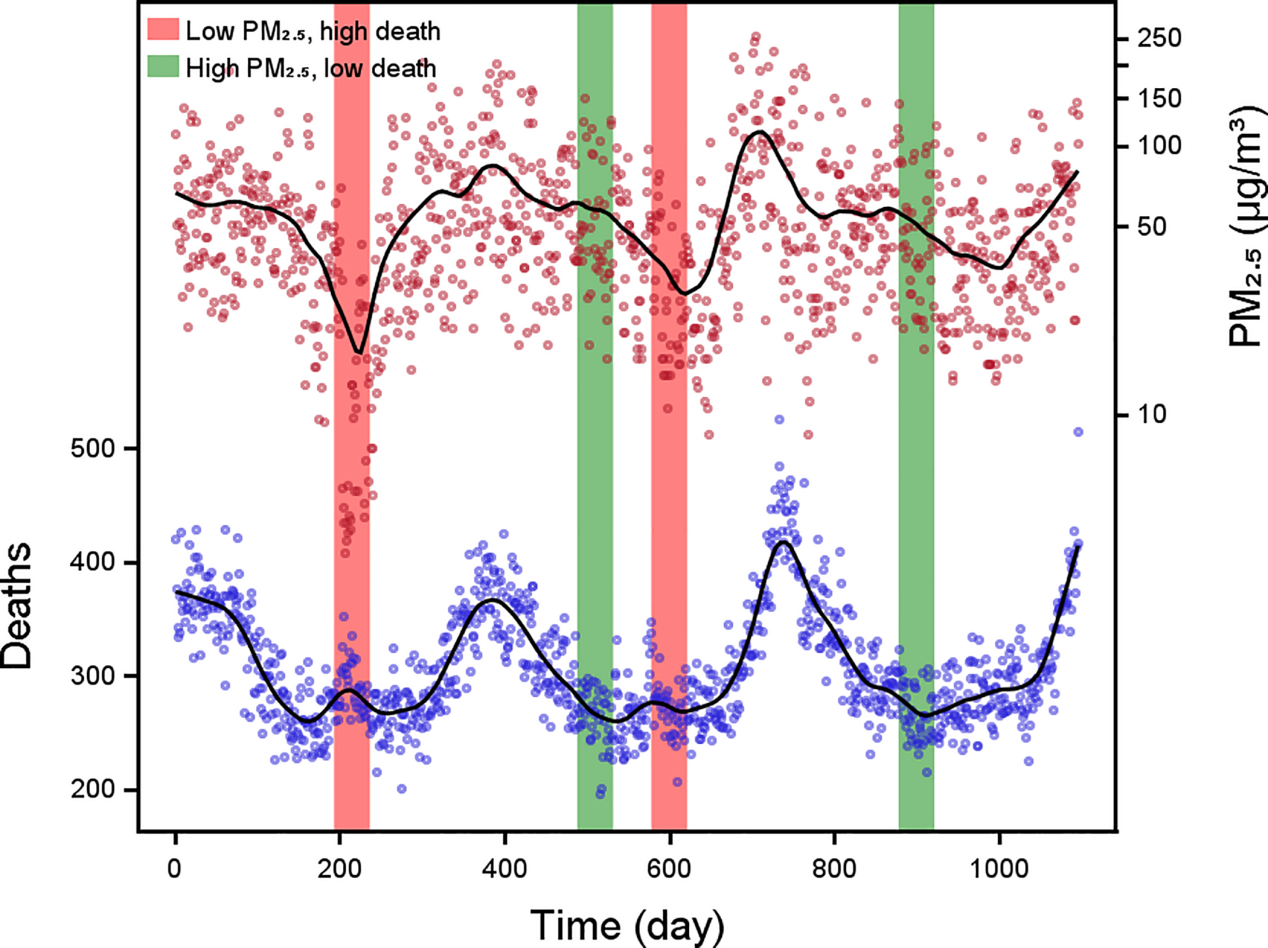
Time trends of daily PM_2.5_ concentrations and deaths.

There were no missing values in the meteorological variables and PM_2.5_ concentrations were missing only in five days in 2012. Generally, the daily average PM_2.5_ concentrations and daily death counts shared the similar temporal trend, i.e. high values presented in cold season and low values in warm season (Fig 1). There were however also opposite trends, i.e. low PM_2.5_ concentrations accompanied with more deaths within a time window of 30 days (indicated by red bands in Fig 1) and high PM_2.5_ concentrations accompanied with less deaths within a time window of 30 days (indicated by green bands in Fig 1), suggesting that the effects of PM_2.5_ on mortality might be modified by weather conditions.

The mean of daily deaths in Shanghai was 307 and the median was 294 during the study period (Table 2). Ambient PM_2.5_ exposure in Shanghai was relatively high with a daily mean of 55.0 μg/m^3^ and median of 45.5 μg/m^3^, higher than the upper limit (35 μg/m^3^) of the level 1 Chinese Ambient Quality Standards [81]. The climate in Shanghai is mild, and generally warm and humid with four distinct seasons. The average annual temperature in Shanghai is 17.2°C, with about 1190 mm of precipitation annually. The detailed daily meteorological conditions are shown in Table 2.

**Table 2:** Descriptive statistics of daily deaths, ambient PM_2.5_ concentrations and meteorological factors in Shanghai, China (2012–2014).

	Mean ± SD	n	Percentiles				
			Min	P_25_	P_50_	P_75_	Max
Daily deaths							
Overall	307±51	1096	196	269	294	339	526
January	390±40	93	316	360	388	415	526
February	358±29	85	292	339	362	371	470
March	334±35	93	256	309	334	358	429
April	298±29	90	227	279	297	319	373
May	275±24	93	196	260	277	293	330
June	259±24	90	215	240	256	276	332
July	278±26	93	231	261	274	294	352
August	275±24	93	207	261	273	285	336
September	274±24	90	201	259	274	289	332
October	277±24	93	225	262	271	292	341
November	301±23	90	249	282	302	318	363
December	366±40	93	284	345	361	387	515
PM_2.5_ (μg/m^3^)							
Overall	55.0±38.6	1091	3.0	29.4	45.5	68.7	447.5
January	78.6±47.3	93	17.5	41.4	58.2	106.2	201.0
February	54.6±33.5	85	8.4	29.4	45.6	72.4	183.0
March	62.5±35.4	93	18.2	37.4	56.6	78.2	191.3
April	56.9±21.4	88	16.1	43.3	55.2	66.6	144.4
May	59.2±29.7	90	18.2	37.7	50.2	70.2	151.0
June	46.2±27.9	90	9.3	23.1	38.0	59.0	127.5
July	38.5±24.1	93	3.0	20.8	39.0	50.2	119.2
August	29.2±18.1	93	4.2	14.0	25.3	39.0	78.2
September	35.7±23.3	90	12.6	19.6	29.7	43.9	125.5
October	43.0±29.6	93	8.4	23.5	36.6	50.2	204.3
November	66.6±40.0	90	21.0	36.6	55.0	86.8	214.0
December	88.2±62.1	93	13.3	54.2	74.4	102.2	447.5
Meteorological factors							
Temperature (°C)	17.2±9.0	1096	-1.2	8.8	18.2	24.3	35.0
Barometric Pressure (kPa)	101.6±0.9	1096	99.5	100.8	101.6	102.3	103.8
Relative Humidity (%)	70.3±12.6	1096	30	62	72	80	98
Wind speed (m/s)	2.80±0.97	1096	0.6	2.1	2.7	3.4	8.6
Precipitation (mm)	3.26±10.35	1096	0	0	0	1.1	195.3
Sunshine (hour)	4.70±3.95	1096	0	0	4.8	8.2	12.9

SD, standard deviation); Px, x^th^ percentiles; Min, minimum; Max, maximum

### Extreme weather conditions

The days with extreme weather conditions were summarized in Table 3. In total, there were 570 days that had at least one extreme weather condition during the study period. There were 181 and 35 days that had two or more extreme weather conditions, respectively. In general, cold and hyperbaria days were the most frequent (60 of 1096 days) and followed by hot and hypobaria days (40 of 1096 days).

**Table 3:** Number of the days with two or more extreme meteorological conditions.

	Hot n = 109	Cold n = 109	Hyperbaria n = 107	Hypobaria n = 105	Humid n = 101	Dry n = 103	Windy n = 100	Windless n = 94
Cold								
Hyperbaria		60						
Hypobaria	40							
Humid				13				
Dry	16	18	12	9				
Windy	14	8	7	22	11	6		
Windless	4	20	11	8	17			

### Feature of the synoptic weather types

The clustering analysis based on the six PCs from the PCA categorized the 1096 days into six synoptic weather types (SWTs). The meteorological characteristics of the six SWTs are show in Table 4.

**Table 4:** Meteorological characteristics and PM_2.5_ concentrations of the six synoptic weather types.

	Number of days	Pressure (kPa)	Temperature (°C)	Humid (%)	Precipitation (mm)	Wind speed (m/s)	Sunshine (hour)	PM_2.5_ (μg/m^3^)
Hot dry	167	100.6±0.4	28.4±4.0	62.0±10.2	1.25±4.55	3.41±0.91	8.79±2.76	41.2±29.3
Warm humid	214	100.8±0.4	23.8±3.8	79.9±6.9	4.11±8.28	2.24±0.63	2.25±32.77	49.5±30.1
Cold dry	158	102.4±0.4	8.0±5.1	60.8±13.2	0.98±3.43	2.82±0.94	5.45±3.39	82.8±50.6
Moderate dry	225	101.7±0.3	18.5±3.8	66.4±10.8	0.32±1.35	2.68±0.68	6.67±3.30	49.0±30.4
Moderate humid	107	101.1±0.6	19.1±6.1	82.3±8.3	17.28±25.26	3.83±1.17	8.99±1.76	40.4±25.1
Cold humid	225	102.5±0.4	6.7±3.2	72.0±9.6	1.81±4.39	2.48±0.82	3.32±3.36	63.5±42.9

According to the meteorological characteristics shown in Table 4, we summarized the features of the six SWTs as:

- Hot dry (HT): the hottest and dry weather type, with sunny and clear sky, relative windy;
- Warm humid (WH): warm, moist, the cloudiest and unstable weather often bring rain showers;
- Cold dry (CD): cold and driest weather type, often cloudy with less precipitation;
- Moderate dry (MD): mild, sunny and clear sky with the least precipitation;
- Moderate humid (MH): a relative rare weather type, mild and the sunniest, unstable often bring intense fall;
- Cold humid (CH): moist and the coldest weather type, stable, most cloudy but with little precipitation.

**Fig 2:**
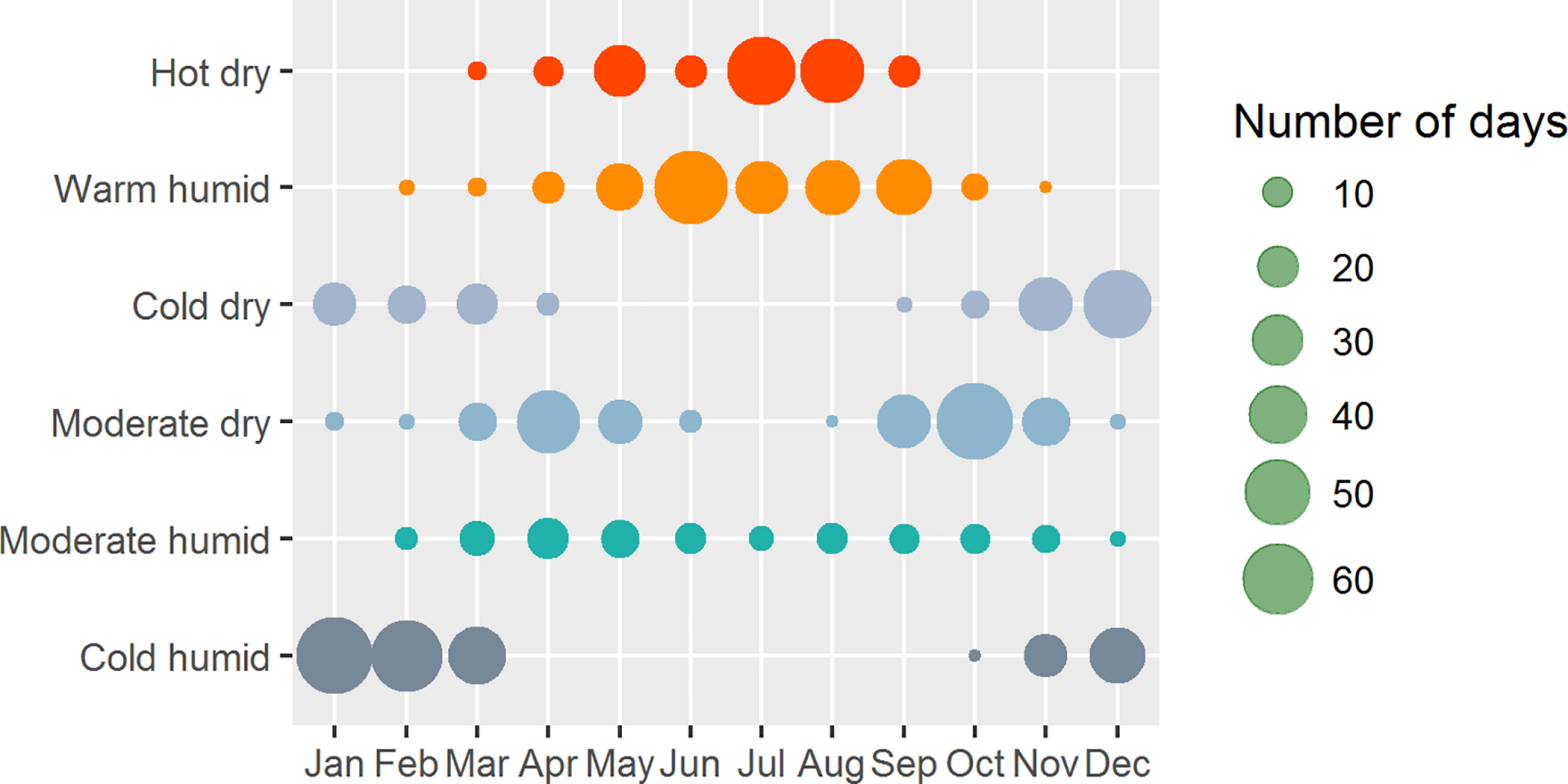
Distribution of the six synoptic weather types during a year.

The total numbers of days of the six SWTs during the study period by the twelve calendar months are shown in Fig 2. In general, cold humid, warm humid and hot dry days account for more than half (55%) of the days in Shanghai.

### MCMC convergence, representativeness, dependency and efficiency

The 97.5% Gelman-Rubin bounds of all the parameters are smaller than 1.01, which indicate the adequate convergence of the Markov chains. The trajectories of parameters reveal that the chains take a few hundred steps to converge to the same region of the parameters and are overlapping fairly and smoothly, suggesting good representativeness. The posterior autocorrelation coefficients of all the parameters after lag 5 are smaller than 0.1, which indicate good mixing and high independency among the Markov chain samples. Most parameters have efficiency higher than 0.6 and adequate ESS (>1000) after 5000 iterations for estimating the posterior density. Example diagnostic results (except for trace plots) of the GAM for PM_2.5_ and the extreme weather conditions without interaction terms are shown in S1–S3 Tables.

### Fitness of the smoothing splines for the GAM

**Fig 3:**
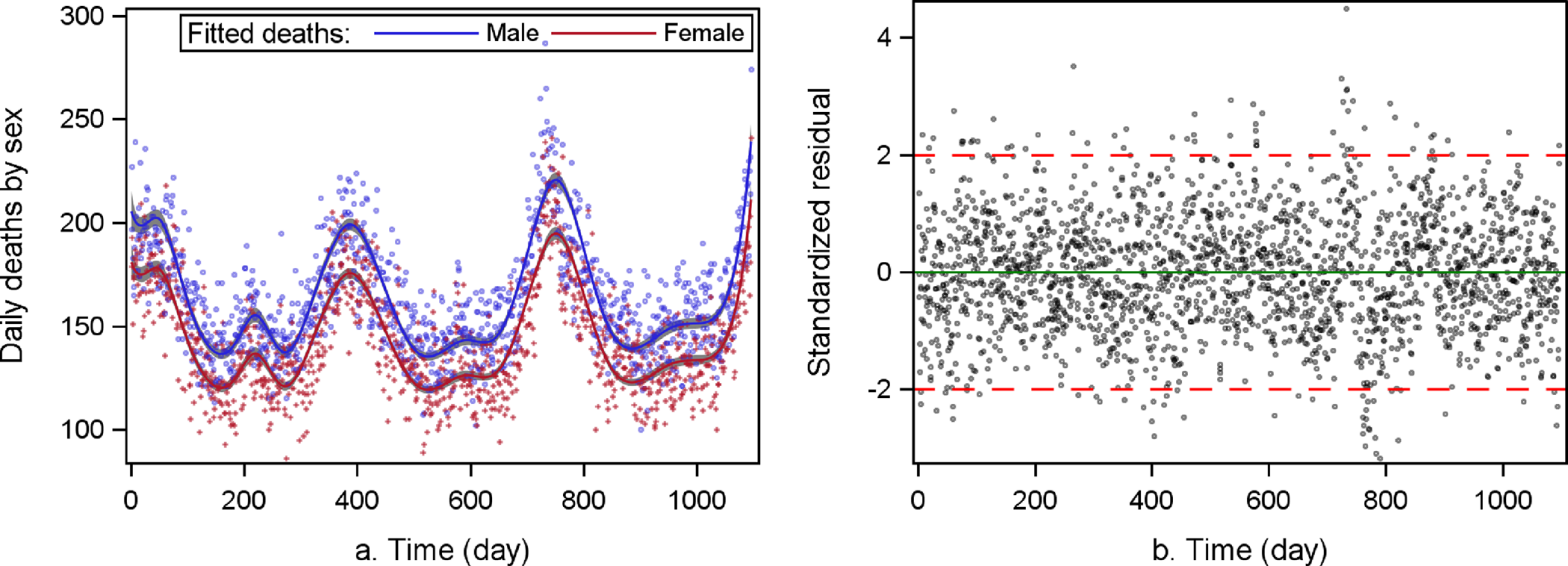
(a) Predicted deaths with 95% equal-tail Bayesian credible intervals, controlling for PM_2.5_ concentrations, sex and synoptic weather types; (b) Standardized residuals.

Fig 3A shows the predicted daily deaths by GAM, after controlling for PM_2.5_, sex and SWTs. The smoothing cubic B-splines fit the time trend very well and more than 95% of the standardized residuals are located in the range of ±2 (Fig 3B). Among equal-tail 95% CrIs of parameters of the smoothing splines, only three or four out of 17 include 0 (example results of GAM for PM_2.5_ and the extreme weather conditions without interaction terms are shown in S4 Table).

### Effects of PM_2.5_ and extreme weather conditions on non-accidental mortality

The effects of PM_2.5_ and the extreme weather conditions on non-accidental mortality are shown in Table 5. Without considering interactions between PM_2.5_ and extreme weather conditions, per 10 μg/m^3^ increase in PM_2.5_ concentration was associated with 0.31 (95% CrI: 0.22, 0.40) percent increase in mortality. Hot, hypobaria and windy days had statistically significant positive associations with mortality, whereas no effect was noted for cold, hyperbaria, humid, dry and windless days. The greatest effect of the extreme weather conditions was found for hot days, where the daily mortality might increase 6.41 (95% CrI: 4.93, 7.96) percent.

**Table 5:** Effects of PM_2.5_, extreme weather conditions and demographic characteristics on non-accidental mortality.

Variables	Percent increase in mortality (95% CrI)	
	Model without interaction	Model with interaction
PM_2.5_ (per 10 μg/m^3^)	0.31 (0.22, 0.40)	0.27 (0.13, 0.41)
Hot	6.41 (4.93, 7.96)	3.59 (1.22, 6.13)
Cold	0.87 (-0.41, 2.07)	0.02 (-2.36, 2.68)
Hyperbaria	0.46 (-0.85, 1.80)	0.73 (-1.77, 3.19)
Hypobaria	1.52 (0.19, 2.87)	-1.55 (-4.05, 1.05)
Humid	0.73 (-0.48, 1.98)	1.41 (-0.36, 3.19)
Dry	-0.75 (-1.91, 0.50)	-4.80 (-7.76, -2.07)
Windy	2.58 (1.29, 3.96)	3.75 (1.74, 5.85)
Windless	-0.60 (-1.91, 0.64)	0.54 (-2.11, 2.96)
Interactions		
PM_2.5_×Hot		0.50 (0.08, 0.95)
PM_2.5_×Cold		0.12 (-0.17, 0.40)
PM_2.5_×Hyperbaria		-0.02 (-0.33, 0.29)
PM_2.5_× Hypobaria		0.62 (0.16, 1.14)
PM_2.5_×Humid		-0.12 (-0.36, 0.10)
PM_2.5_×Dry		0.59 (0.21, 1.00)
PM_2.5_×Windy		-0.22 (-0.66, 0.19)
PM_2.5_×Windless		-0.15 (-0.41, 0.12)
Female	47.68 (44.55, 51.00)	47.60 (44.49, 50.85)
Age		
0–14 years	-98.81 (-98.87, -98.75)	-98.81 (-98.88, -98.74)
15–39 years	-99.32 (-99.34, -99.30)	-99.32 (-99.34, -99.29)
40–64 years	-94.43 (-94.51, -94.33)	-94.42 (-94.52, -94.34)
65+ years (Ref)		
Occupation		
Governmental	-97.78 (-97.87, -97.69)	-97.78 (-97.86, -97.70)
Professional	-76.63 (-76.94, -76.32)	-76.62 (-76.90, -76.32)
Administrative	-69.83 (-70.21, -69.49)	-69.82 (-70.18, -69.47)
Business	-73.53 (-73.84, -73.23)	-73.55 (-73.87, -73.23)
Agriculture	-37.26 (-37.84, -36.71)	-37.25 (-37.77, -36.69)
Manufactory (Ref)		
Military	-99.84 (-99.86, -99.81)	-99.84 (-99.86, -99.81)
Others	-97.43 (-97.53, -97.34)	-97.43 (-97.52, -97.32)
Preschool	-99.15 (-99.19, -99.10)	-99.15 (-99.20, -99.09)
Students	-99.73 (-99.75, -99.70)	-99.73 (-99.76, -99.69)
Jobless	-77.62 (-77.93, -77.34)	-77.63 (-77.90, -77.35)
Day of week		
Sunday (Ref)		
Monday	1.67 (0.45, 3.00)	1.73 (0.27, 3.04)
Tuesday	0.68 (-0.56, 1.95)	0.70 (-0.52, 2.04)
Wednesday	0.93 (-0.33, 2.24)	0.89 (-0.35, 2.11)
Thursday	-0.01 (-1.24, 1.32)	0.07 (-1.19, 1.35)
Friday	0.05 (-1.14, 1.41)	0.03 (-1.17, 1.24)
Saturday	0.09 (-1.08, 1.47)	0.04 (-1.24, 1.26)
Smoking rate	2.01 (1.95, 2.08)	2.01 (1.95, 2.08)

When considering interactions between PM_2.5_ and extreme weather conditions, the effect of PM_2.5_ diminished slightly (percent increase = 0.27, 95% CrI: 0.13, 0.41). However, the effects of extreme weather conditions had significant changed with the strongest but a reverse association found in dry days (percent increase = –4.80, 95% CrI: = –7.76, –2.07). Statistically significant interactions were found between PM_2.5_ and hot, hypobaria and dry days. All of the three interactions are positive interactions. Even in dry days, per 10 μg/m^3^ increase in PM_2.5_ concentration might result in about 0.86 (= 0.27 + 0.59) percent increase in mortality, although the overall effect in dry days is lowest.

### Effects of PM_2.5_ and synoptic weather types on non-accidental mortality

The effects of PM_2.5_ and the SWTs on non-accidental mortality are shown in Table 6. Without considering the interactions between PM_2.5_ and SWTs, per 10 μg/m^3^ increase in PM_2.5_ concentration was associated with 0.35 (96% CrI: 0.26, 0.44) percent increase in mortality. Compared to cold humid SWT, hot dry SWT had the greatest effect on mortality (percent increase in mortality = 7.09, 95% CrI: 5.18, 9.14), followed by moderate humid SWT (percent increase = 5.36, 95% CrI: 3.61, 7.08), and warm humid SWT (percent increase = 2.18, 95% CrI: 0.41, 4.11). By contrast, cold dry SWT had the smallest effect (percent increase = –1.98, 95% CrI: –3.15, –0.85).

**Table 6:** Effects of PM_2.5_, synoptic weather types and demographic characteristics on non-accidental mortality.

Variable	Percent increase in mortality (95% CrI)	
	Model without interaction	Model with interaction
PM2.5	0.35 (0.26, 0.44)	0.26 (0.10, 0.43)
Synoptic weather types		
Hot dry	7.09 (5.18, 9.14)	1.51 (-1.42, 4.52)
Warm humid	2.18 (0.41, 4.11)	-0.32 (-2.78, 2.37)
Cold dry	-1.98 (-3.15, -0.85)	-1.84 (-3.83, 0.23)
Moderate dry	1.94 (0.48, 3.37)	2.78 (0.53, 5.13)
Moderate humid	5.36 (3.61, 7.08)	4.37 (1.49, 7.32)
Cold humid (Ref)		
Interactions		
PM_2.5_×Hot dry		1.02 (0.62, 1.40)
PM_2.5_× Warm humid		0.38 (0.05, 0.70)
PM_2.5_×Cold dry		0.00 (-0.23, 0.23)
PM_2.5_×Moderate dry		-0.16 (-0.47, 0.14)
PM_2.5_×Moderate humid		0.16 (-0.27, 0.63)
PM_2.5_×Cold humid (Ref)		
Female	47.74 (44.6, 51.20)	47.57 (43.84, 50.83)
Age		
0–14 years	-98.81 (-98.88, -98.74)	-98.81 (-98.88, -98.74)
15–39 years	-99.32 (-99.34, -99.29)	-99.32 (-99.34, -99.30)
40–64 years	-94.43 (-94.51, -94.34)	-94.42 (-94.52, -94.34)
65+ years (Ref)		
Occupation		
Governmental	-97.78 (-97.87, -97.69)	-97.78 (-97.87, -97.70)
Professional	-76.62 (-76.91, -76.32)	-76.64 (-76.93, -76.34)
Administrative	-69.81 (-70.13, -69.46)	-69.82 (-70.20, -69.42)
Business	-73.55 (-73.84, -73.23)	-73.55 (-73.90, -73.24)
Agriculture	-37.24 (-37.81, -36.64)	-37.25 (-37.79, -36.69)
Manufactory (Ref)		
Military	-99.84 (-99.86, -99.81)	-99.84 (-99.86, -99.81)
Others	-97.43 (-97.52, -97.33)	-97.43 (-97.52, -97.35)
Preschool	-99.15 (-99.20, -99.09)	-99.15 (-99.20, -99.09)
Students	-99.73 (-99.76, -99.70)	-99.73 (-99.76, -99.70)
Jobless	-77.62 (-77.91, -77.33)	-77.63 (-77.93, -77.35)
Day of week		
Sunday (Ref)		
Monday	1.88 (0.63, 3.24)	1.91 (0.63, 3.27)
Tuesday	0.92 (-0.34, 2.24)	0.88 (-0.33, 2.12)
Wednesday	0.95 (-0.39, 2.20)	0.98 (-0.30, 2.17)
Thursday	0.24 (-0.97, 1.56)	0.31 (-0.92, 1.57)
Friday	-0.10 (-1.35, 1.13)	-0.10 (-1.30, 1.22)
Saturday	0.07 (-1.19, 1.43)	0.06 (-1.14, 1.32)
Smoking rate	2.02 (1.95, 2.09)	2.01 (1.94, 2.09)

When considering the interactions between PM_2.5_ and SWTs, the effects of SWTs on mortality shown significant changed, with the highest effect found in moderate humid SWT (percent increase = 4.37, 95% CrI: 1.49, 7.32) and followed by moderate dry SWT (percent increase = 2.78, 95% CrI: 0.53, 5.13). Statistically significant interactions were found between PM_2.5_ and hot dry and warm humid SWTs. Considering the interaction with weather type, the smallest effect of PM_2.5_ on mortality was found in moderate dry SWT (percent increase = 0.10, but not statistically significant, 95% CrI: -0.37, 0.29) and the greatest effect was found in hot dry SWT (percent increase = 1.28, 95% CrI: 0.72, 1.83), followed by warm humid SWT (percent increase = 0.64, 95% CrI: 0.15, 1.13).

### Effects of demographic characteristics and smoking on non-accidental mortality

Although the effects of demographic characteristics and smoking on non-accidental mortality were out of the main interest of this study, they are similar in both the extreme weather condition models and the SWT models. After controlling for age, smoking rate and occupations, the mortality risk was about 48% higher in women than in men. Compared with other occupations, people worked in governmental agencies, studied in schools and serviced in military had the lowest risk of non-accidental mortality.

### Sensitivity analysis

We performed a sensitivity analysis using the estimates from Chen’s study [82] as the informative normal prior mean in the Bayesian reference but did not find detectable change in the results.

## Discussion

### Modification of the association between PM_2.5_ and non-accidental mortality by weather conditions

A substantial number of time-series studies have demonstrated an association between mortality and exposure to PM_2.5_ air pollution while controlling for confounding factors that also vary over time, such as weather and season [83]. The usual analytic approach to control for weather is to include weather variables, typically temperature and humidity, in regression models that evaluate the effect of air pollution on mortality. However, an inappropriate set of weather variables and the correlations among weather variables as well as between weather variables and air pollution [84, 85] could bias the estimate of the effect of air pollution on mortality. One alternate approach for controlling the potential confounding by weather is to use the synoptic categorization of weather. Our study evaluated the applicability of SWTs to assess the short term association between PM_2.5_ and mortality in Shanghai, China. We found statistically significant association between PM_2.5_ concentration and non-accidental mortality in Shanghai, China, i.e. per 10 μg/m^3^ increase in daily average PM_2.5_ concentration alone corresponds to 0.26–0.35% increase in daily non-accidental mortality. The increased risk is slightly higher than the 0.22% increase in a recently published paper by Chen et al., who conducted a nationwide analysis using the PM_2.5_ concentration data between 2013 and 2015 in 272 Chinese cities [82]. The risk increase found in the present study is similar to Lippmann et al. based on a recent multicity study in 150 U.S cities[86], but appreciable smaller than results found in other multicity studies in Europe and North America, where the increased risks for non-accidental mortality due to all causes ranged from 0.55% to 1.18% [87–91].

In our study, PM_2.5_ levels were higher in cold days than in warm days and the same variation was also found for mortality, i.e. more non-accidental deaths in winter than in summer (Table 2). However, higher mortality was found in extreme hot days compared to extreme cold days when adjusting for other demographic variables. We found the association between PM_2.5_ and mortality to be modified by weather conditions. The strongest interactions were found between PM_2.5_ and hot, dry and hypobaria days. Because of the inverse relationship between temperature and barometric pressure and frequent co-occurrence of hot and hypobaria days (Table 3), it suggests that PM_2.5_ poses higher risk in hot days than in other days in Shanghai. As expected, extreme hot weather had a positive association with daily mortality and a synergistic action with PM_2.5_. No statistically significant association and interaction were however found for extreme cold weather. One reason might be the subtropical climate of Shanghai with an average temperature over than 0°C even in the coldest months. Although hypobaria condition alone had a positive association with daily mortality, which is consistent with the findings in another Chinese city [92], its effect was reversed when considering its interaction with PM_2.5_. No statistically significant association was found for extreme humid or extreme dry weather alone. When taking into account interactions, hypobaria and extreme dry weather had statistically significant synergistic reactions with PM_2.5_. The positive interactions between hypobaria and extreme dry weather and PM_2.5_ might be due to the low atmospheric pressure and humidity that may induce hypoxia and excessive dehydration of nasal passages and the upper respiratory tract, leading to increased risk of severe cerebrovascular and cardiovascular diseases [93, 94] as well as microbial and viral infections[95].

Besides, we are more interested in the PM_2.5_-mortality relationships when we consider the effects from weather variables as a whole. To what we did not expect, statistically significant positive associations between PM_2.5_ and mortality were found for favorable SWTs, i.e. moderate day and moderate humid weather types, when including the interaction terms in the models. The higher excessive mortality attributable to PM_2.5_ in stable and comfortable weather conditions might suggest that the effect of air pollution is more pronounced in milder weathers than in extreme weathers. The reason might be the lower baseline risk in comfortable weather conditions, which results the larger relative risk associated with certain exposure in comfortable weather conditions compared to unfavorable ones. But we should also note that human behavior might change in different SWTs, for example, people tend to reduce outdoor activities in poor or extreme weathers, leading to reduced exposure to outdoor air pollution [96]. On the contrary, people tend to spend more time outside on pleasant days, potentially leading to increased likelihood of exposure to PM_2.5_ and to a larger dose of PM_2.5_.

Although Samet [97] and Pope et al. [98] found little evidence that weather conditions modified the effect of air pollution, Rainham [42], Vanos [99–102] and Vaneckova [103] et al. reported that change of synoptic type could alter the strength of pollutant associations with mortality and statistically significant association of pollutant with mortality was only noted during summer (warm, hot and dry) weather and stronger association was noted for fair synoptic types. In general, our findings are consistent with those from Canada and Australia.

### Bayesian generalized additive model analysis

We used a full Bayesian method for GAM analysis in our study to fully account for the uncertainty of the underlying parameters. The method deems that data are observed from the realized sample and underlying parameters are unknown and can be described probabilistically. In addition, when study investigators had a strong *a priori* belief that the dose-response relationship between PM_2.5_ and mortality is non-decreasing (not necessarily linear) and wanted an estimate consistent with this assumption, Bayesian GAM is a better alternative to a frequentist method. Furthermore, when studying the potential effect modification by weather, the days were distributed into SWTs with loss of precision for estimates in these categories. Consequently, there is a potential risk to over-interpret variation in the parameters [97]. The Bayesian method used in the study is effective to avoid this problem by drawing sufficient large MCMC samples to make the reference arbitrarily accurate [59].

Although at the cost of computational time, the Bayesian GAM offers significant accuracy improvements compared to conventional methods [104]. We should keep in mind that priors should be rationally and honestly derived. They can be weak or strong. When the prior is weak, the prior distribution will be wide and the likelihood will be more influential in creating the posterior distribution. Conversely, when the prior is strong, the prior distribution will be narrow and the likelihood will be less influential in creating the posterior. It should be clear the one key feature of the prior is the ability to quantify our uncertainty. The posterior can be thought of as a compromise between the prior and the likelihood.

### Strengths and limitations

There are some strengths in our study. First, we used individual-level demographic characteristics and socioeconomic factors in our study and adjusted for these potentially important confounders, especially smoking, in our analysis. Compared with most of the previous time-series studies of PM_2.5_ and mortality, which were based on population-level aggregated data, our study minimized the ecological fallacy. Second, as pointed by Bernstein–von Mises theorem [105], in large data samples, the posterior distribution is independent of the prior distribution and, therefore, Bayesian and likelihood-based inferences could yield essentially the same results. Third, we used SWTs rather than individual meteorological variables to control for the weather conditions, which optimally create categories with days similar to one another in weather variables and different from days in other categories. The major advantage of the synoptic approach is that it examines the biological effect as the organism’s response to ambient atmospheric conditions rather than to individual variables such as temperature and atmospheric pressure [42]. Last but not least, we examined weather-air pollution interactions with mortality. Improved knowledge of the modified effects of PM_2.5_ on mortality by weather conditions is vital for the medical society, policy makers and community leaders to implement proper intervention strategies [23, 101].

There are also some limitations in our study. First, like most of the epidemiological studies on air pollution and mortality, only city-wide average PM_2.5_ concentrations were available in our study, instead of personal exposure to PM_2.5_. However, Janssen et al. reported high correlation between personal PM exposure and outdoor PM concentrations longitudinally [106]. Zeger et al explored the issue in more details and showed that the association could only be underestimated when using city level PM concentration as a proxy for personal exposure level [107]. Second, because main purpose of our study is to examine the application of Bayesian method in GAM in investigating the interactions between PM_2.5_ and weather conditions, no lag effects were evaluated for PM_2.5_ and weather conditions in the present study. The issues of single day lags and distributed lags are left for future study. Third, as an exploratory study, no cause-specific association was evaluated. According to the WHO HRAPIE impact assessment for long-term exposure to PM_2.5_ and non-accidental deaths due to all causes and the American Cancer Society Cohort Study, the relative risk for mortality was about 1.06 per 10 μg/m^3^ increment of the annual average PM_2.5_ concentration [108–110]. The assumption of 6% increment in cardio-respiratory mortality per 10 μg/m^3^ increment in PM_2.5_ concentration has been used in many health impact assessments. It is much higher than the risk for non-accidental mortality due to all causes that we found in the present study and the results published in the latest multi-city study in China [82]. Further studies on cause-specific mortality are warranted. Because the methodology for the cause-specific analysis would be the same, we have planned to perform the same risk assessment for respiratory, cerebrovascular and cardiovascular mortalities in the future. Last, the association was only based on one-pollutant model and the effects from unmeasured co-exposure to other air pollutants might have been masked in smoothing functions. In the future, the similar method will be employed for two- or multiple-pollutant models.

## Conclusions

We found that the effect of PM_2.5_ on non-accidental mortality differed under specific extreme weather conditions and SWTs. Models both with and without interactions between PM_2.5_ and weather display statistically significant increase in mortality due to PM_2.5_. Our results correspond well to the evaluations of air pollution, weather types and mortality in previous studies [42, 99–103]. Given the statistically significant interactions between PM_2.5_ and weather and climate and pollution challenges, adequate policies and public health actions are needed, taking into account the interrelationship between the two hazardous exposures. Environmental policy makers should consider the application of the synoptic approach in decision making and prevention activities to ameliorate the adverse effects from air pollution.

## Supporting information

S1 Fig Map of Shanghai city. Yellow part is center urban districts and white part is suburban districts.
(TIF)

S1 Table Gelman-Rubin diagnostics for PM_2.5_ and extreme weather conditions without interaction. (DOCX)

S2 Table Posterior autocorrelations for PM_2.5_ and extreme weather conditions without interaction. (DOCX)

S3 Table Effective sample sizes (ESS) for PM_2.5_ and extreme weather conditions without interaction. (DOCX)

S4 Table Posterior intervals of parameters of cubic B-splines for PM_2.5_ and extreme weather conditions without interaction. (DOCX)

S5 Table STROBE Statement—Checklist of items that should be included in reports of observational studies. (DOC)

## Abbreviation

PM_2.5_: particulate matters with aerodynamic diameters equal to or less than 2.5 μm
GAM: generalized additive model
GAMM: generalized additive mixed model
PCA: principal component analysis
MCMC: Markov chain Monte Carlo
ESS: effective sample sizes
SWT: synoptic weather type
CrI: credible interval
