# Supplementary material for: Relationship between fine particulate matter, weather condition and daily non-accidental mortality in Shanghai, China: A Bayesian approach
Source: PLoS One. 2017 Nov 9;12(11):e0187933. doi: 10.1371/journal.pone.0187933 (PMC5679525; doi:10.1371/journal.pone.0187933)
Supplement: S1 Table — (DOCX) [file pone.0187933.s002.docx]

**S1 Table.** **Gelman-Rubin diagnostics for PM_2.5_ and extreme weather conditions without interaction**

| Parameter | Estimate | 97.5% Bound |
| --- | --- | --- |
| Intercept | 1.0032 | 1.0071 |
| PM_2.5_ | 1.0007 | 1.0055 |
| Hot | 0.9999 | 1.0040 |
| Cold | 1.0019 | 1.0045 |
| Hyperbaria | 1.0021 | 1.0064 |
| Hypobaria | 0.9996 | 1.0042 |
| Humid | 1.0041 | 1.0071 |
| Dry | 1.0027 | 1.0041 |
| Windy | 1.0013 | 1.0042 |
| Windless | 1.0001 | 1.0042 |
| Female | 0.9995 | 1.0044 |
| 0-14 years | 1.0017 | 1.0051 |
| 15-39 years | 1.0010 | 1.0041 |
| 40-64 years | 1.0017 | 1.0046 |
| Governmental | 1.0047 | 1.0042 |
| Professional | 1.0041 | 1.0043 |
| Administrative | 1.0048 | 1.0042 |
| Business | 1.0038 | 1.0043 |
| Agriculture | 1.0049 | 1.0041 |
| Military | 1.0013 | 1.0053 |
| Others | 1.0031 | 1.0065 |
| Preschool | 1.0105 | 1.0054 |
| Students | 1.0079 | 1.0053 |
| Jobless | 1.0037 | 1.0040 |
| Monday | 1.0006 | 1.0044 |
| Tuesday | 1.0003 | 1.0050 |
| Wednesday | 0.9993 | 1.0042 |
| Thursday | 0.9992 | 1.0041 |
| Friday | 1.0019 | 1.0068 |
| Saturday | 1.0023 | 1.0053 |
| Smoking rate | 0.9994 | 1.0042 |
| B-spline 1 | 1.0033 | 1.0070 |
| B-spline 2 | 1.0029 | 1.0068 |
| B-spline 3 | 1.0030 | 1.0070 |
| B-spline 4 | 1.0030 | 1.0066 |
| B-spline 5 | 1.0029 | 1.0069 |
| B-spline 6 | 1.0031 | 1.0068 |
| B-spline 7 | 1.0029 | 1.0066 |
| B-spline 8 | 1.0035 | 1.0073 |
| B-spline 9 | 1.0026 | 1.0065 |
| B-spline 10 | 1.0031 | 1.0069 |
| B-spline 11 | 1.0028 | 1.0066 |
| B-spline 12 | 1.0033 | 1.0071 |
| B-spline 13 | 1.0026 | 1.0064 |
| B-spline 14 | 1.0028 | 1.0067 |
| B-spline 15 | 1.0032 | 1.0070 |
| B-spline 16 | 1.0027 | 1.0066 |
| B-spline 17 | 1.0016 | 1.0051 |
