# Supplementary material for: Relationship between fine particulate matter, weather condition and daily non-accidental mortality in Shanghai, China: A Bayesian approach
Source: PLoS One. 2017 Nov 9;12(11):e0187933. doi: 10.1371/journal.pone.0187933 (PMC5679525; doi:10.1371/journal.pone.0187933)
Supplement: S2 Table — (DOCX) [file pone.0187933.s003.docx]

**S2 Table.** **Posterior autocorrelations for PM_2.5_ and extreme weather conditions without interaction**

| Parameter | Lag 1 | Lag 5 | Lag 10 | Lag 50 |
| --- | --- | --- | --- | --- |
| Intercept | 0.1574 | -0.0430 | -0.0048 | 0.0641 |
| PM_2.5_ | 0.2820 | 0.0283 | -0.0028 | 0.0034 |
| Hot | 0.1743 | -0.0803 | 0.0264 | 0.0500 |
| Cold | 0.2226 | -0.0308 | 0.0112 | -0.0203 |
| Hyperbaria | 0.1707 | -0.0371 | 0.0504 | 0.0056 |
| Hypobaria | 0.2415 | 0.0390 | -0.0255 | -0.0591 |
| Humid | 0.2127 | -0.0060 | 0.0848 | 0.0130 |
| Dry | 0.2276 | -0.0204 | -0.0388 | 0.0373 |
| Windy | 0.2085 | -0.0466 | 0.0327 | -0.0220 |
| Windless | 0.2371 | -0.0297 | 0.0234 | 0.0745 |
| Female | 0.1644 | -0.0461 | -0.0254 | -0.0219 |
| 0-14 years | 0.2416 | -0.0173 | 0.0078 | 0.0121 |
| 15-39 years | 0.2267 | -0.0124 | -0.0116 | 0.0351 |
| 40-64 years | 0.2410 | -0.0327 | -0.0003 | 0.0179 |
| Governmental | 0.2537 | 0.0006 | 0.0041 | -0.0160 |
| Professional | 0.2353 | -0.0132 | 0.0366 | -0.0272 |
| Administrative | 0.2479 | -0.0007 | 0.0138 | -0.0320 |
| Business | 0.2614 | -0.0039 | 0.0257 | -0.0415 |
| Agriculture | 0.2570 | -0.0167 | 0.0342 | -0.0410 |
| Military | 0.2879 | 0.0078 | -0.0770 | 0.0006 |
| Others | 0.2089 | 0.0032 | -0.0034 | -0.0611 |
| Preschool | 0.1936 | -0.0119 | 0.0588 | 0.0116 |
| Students | 0.2186 | 0.0773 | -0.0059 | 0.0963 |
| Jobless | 0.2709 | -0.0165 | 0.0352 | -0.0155 |
| Monday | 0.1691 | -0.0213 | -0.0210 | -0.0105 |
| Tuesday | 0.2638 | 0.0016 | 0.0343 | -0.0035 |
| Wednesday | 0.1985 | -0.0355 | 0.0300 | -0.0260 |
| Thursday | 0.2078 | 0.0021 | 0.0161 | 0.0360 |
| Friday | 0.2335 | -0.0439 | 0.0211 | 0.0208 |
| Saturday | 0.1883 | -0.0023 | 0.0285 | -0.0035 |
| Smoking rate | 0.1846 | -0.0585 | -0.0173 | -0.0224 |
| B-spline 1 | 0.1534 | -0.0485 | 0.0018 | 0.0600 |
| B-spline 2 | 0.1564 | -0.0512 | 0.0025 | 0.0600 |
| B-spline 3 | 0.1518 | -0.0452 | -0.0007 | 0.0610 |
| B-spline 4 | 0.1608 | -0.0485 | 0.0025 | 0.0618 |
| B-spline 5 | 0.1500 | -0.0483 | 0.0008 | 0.0626 |
| B-spline 6 | 0.1608 | -0.0476 | 0.0004 | 0.0686 |
| B-spline 7 | 0.1500 | -0.0498 | 0.0005 | 0.0600 |
| B-spline 8 | 0.1589 | -0.0505 | 0.0025 | 0.0619 |
| B-spline 9 | 0.1568 | -0.0511 | 0.0019 | 0.0613 |
| B-spline 10 | 0.1542 | -0.0478 | 0.0006 | 0.0618 |
| B-spline 11 | 0.1557 | -0.0479 | 0.0003 | 0.0589 |
| B-spline 12 | 0.1546 | -0.0472 | 0.0022 | 0.0639 |
| B-spline 13 | 0.1547 | -0.0511 | 0.0032 | 0.0584 |
| B-spline 14 | 0.1547 | -0.0461 | 0.0028 | 0.0643 |
| B-spline 15 | 0.1598 | -0.0500 | -0.0026 | 0.0611 |
| B-spline 16 | 0.1476 | -0.0384 | 0.0102 | 0.0590 |
| B-spline 17 | 0.1935 | -0.0664 | -0.0120 | 0.0451 |
