# Supplementary material for: Relationship between fine particulate matter, weather condition and daily non-accidental mortality in Shanghai, China: A Bayesian approach
Source: PLoS One. 2017 Nov 9;12(11):e0187933. doi: 10.1371/journal.pone.0187933 (PMC5679525; doi:10.1371/journal.pone.0187933)
Supplement: S3 Table — (DOCX) [file pone.0187933.s004.docx]

**S3 Table.** **Effective sample sizes (ESS) for PM_2.5_ and extreme weather conditions without interaction**

| Parameter | ESS | Autocorrelation time | Efficiency |
| --- | --- | --- | --- |
| Intercept | 3797.0 | 1.3148 | 0.7606 |
| PM_2.5_ | 4002.0 | 1.9852 | 0.5037 |
| Hot | 2949.0 | 1.4440 | 0.6925 |
| Cold | 3189.0 | 1.4583 | 0.6857 |
| Hyperbaria | 3154.5 | 1.3414 | 0.7455 |
| Hypobaria | 3208.0 | 1.5602 | 0.6409 |
| Humid | 3058.0 | 1.4466 | 0.6913 |
| Dry | 3071.5 | 1.3059 | 0.7658 |
| Windy | 3343.5 | 1.4650 | 0.6826 |
| Windless | 2942.5 | 1.5182 | 0.6587 |
| Female | 3373.5 | 1.3168 | 0.7594 |
| 0-14 years | 2998.0 | 1.2494 | 0.8004 |
| 15-39 years | 3229.0 | 1.6954 | 0.5898 |
| 40-64 years | 3115.0 | 1.5680 | 0.6378 |
| Governmental | 3736.5 | 1.5849 | 0.6309 |
| Professional | 3273.0 | 1.5585 | 0.6416 |
| Administrative | 4153.0 | 1.6349 | 0.6116 |
| Business | 3870.0 | 1.6280 | 0.6143 |
| Agriculture | 3356.5 | 1.4954 | 0.6687 |
| Military | 3904.5 | 1.6991 | 0.5885 |
| Others | 3816.0 | 1.4822 | 0.6747 |
| Preschool | 3891.5 | 1.6678 | 0.5996 |
| Students | 3808.5 | 1.5485 | 0.6458 |
| Jobless | 3917.5 | 1.6050 | 0.6230 |
| Monday | 3783.5 | 1.3382 | 0.7473 |
| Tuesday | 3929.0 | 1.5276 | 0.6546 |
| Wednesday | 3783.5 | 1.2039 | 0.8306 |
| Thursday | 3931.5 | 1.2919 | 0.7740 |
| Friday | 3854.0 | 1.4897 | 0.6713 |
| Saturday | 3892.0 | 1.2806 | 0.7809 |
| Smoking rate | 3821.5 | 1.3103 | 0.7632 |
| B-spline 1 | 3884.5 | 1.2849 | 0.7783 |
| B-spline 2 | 3895.0 | 1.3128 | 0.7617 |
| B-spline 3 | 3818.5 | 1.2764 | 0.7835 |
| B-spline 4 | 3905.5 | 1.3216 | 0.7567 |
| B-spline 5 | 3789.0 | 1.2726 | 0.7858 |
| B-spline 6 | 4003.0 | 1.3215 | 0.7567 |
| B-spline 7 | 3524.0 | 1.2717 | 0.7863 |
| B-spline 8 | 3797.0 | 1.2973 | 0.7708 |
| B-spline 9 | 4002.0 | 1.2847 | 0.7784 |
| B-spline 10 | 2949.0 | 1.3084 | 0.7643 |
| B-spline 11 | 3189.0 | 1.2871 | 0.7769 |
| B-spline 12 | 3154.5 | 1.2837 | 0.7790 |
| B-spline 13 | 3208.0 | 1.3094 | 0.7637 |
| B-spline 14 | 3058.0 | 1.2802 | 0.7811 |
| B-spline 15 | 3071.5 | 1.3195 | 0.7578 |
| B-spline 16 | 3343.5 | 1.2491 | 0.8006 |
| B-spline 17 | 2942.5 | 1.4188 | 0.7048 |
