# Supplementary material for: Relationship between fine particulate matter, weather condition and daily non-accidental mortality in Shanghai, China: A Bayesian approach
Source: PLoS One. 2017 Nov 9;12(11):e0187933. doi: 10.1371/journal.pone.0187933 (PMC5679525; doi:10.1371/journal.pone.0187933)
Supplement: S4 Table — (DOCX) [file pone.0187933.s005.docx]

**S4 Table.** **Posterior intervals of parameters of cubic B-splines for PM_2.5_ and extreme weather conditions without interaction**

| Parameter | Alpha | Equal-Tail Interval | |  | HPD Interval | |
| --- | --- | --- | --- | --- | --- | --- |
| B-spline 1 | 0.050 | -1.2340 | 0.0395 |  | -1.2503 | 0.00572 |
| B-spline 2 | 0.050 | -0.9261 | 0.0954 |  | -0.9283 | 0.0909 |
| B-spline 3 | 0.050 | -1.2912 | -0.1576 |  | -1.2562 | -0.1412 |
| B-spline 4 | 0.050 | -1.4793 | -0.4030 |  | -1.4836 | -0.4145 |
| B-spline 5 | 0.050 | -1.2532 | -0.1451 |  | -1.2001 | -0.1084 |
| B-spline 6 | 0.050 | -1.4422 | -0.3565 |  | -1.4422 | -0.3611 |
| B-spline 7 | 0.050 | -1.2119 | -0.1004 |  | -1.1647 | -0.0789 |
| B-spline 8 | 0.050 | -0.9412 | 0.1506 |  | -0.8944 | 0.1639 |
| B-spline 9 | 0.050 | -1.5313 | -0.4148 |  | -1.4897 | -0.4094 |
| B-spline 10 | 0.050 | -1.3864 | -0.2923 |  | -1.3427 | -0.2590 |
| B-spline 11 | 0.050 | -1.4339 | -0.3393 |  | -1.4076 | -0.3348 |
| B-spline 12 | 0.050 | -0.7647 | 0.3245 |  | -0.7238 | 0.3406 |
| B-spline 13 | 0.050 | -1.5462 | -0.4361 |  | -1.5075 | -0.4136 |
| B-spline 14 | 0.050 | -1.4291 | -0.3520 |  | -1.4182 | -0.3489 |
| B-spline 15 | 0.050 | -1.3366 | -0.2312 |  | -1.2970 | -0.2103 |
| B-spline 16 | 0.050 | -1.4187 | -0.3266 |  | -1.3712 | -0.3108 |
| B-spline 17 | 0.050 | 0.1319 | 1.3346 |  | 0.1701 | 1.3427 |
